# Supplementary material for: Human IgG Fc-engineering for enhanced plasma half-life, mucosal distribution and killing of cancer cells and bacteria
Source: Nat Commun. 2024 Mar 7;15:2007. doi: 10.1038/s41467-024-46321-9 (PMC10920689; doi:10.1038/s41467-024-46321-9)
Supplement: Supplementary file 1 — Supplementary Information [file 41467_2024_46321_MOESM1_ESM.pdf]

Supplementary Information

SI Text 1:

The FcRn:IgG1 Fc binding interface is largely hydrophobic in nature which together with formation of hydrogen bonds and salt bridges constitute the major forces of attraction. Regarding pH-dependency, IgG1 Fc residue H310 is considered crucial as it becomes positively charged at acidic pH (pH 6.0) and forms a strong hydrogen bond with FcRn residue E115[21]. To provide a rationale for the REW amino acid substitutions, we took advantage of a co-crystal structure of human FcRn in complex with IgG1 Fc[21] solved at acidic pH (5.2) and introduced the Q311R, M428E and N434W substitutions using PyMOL (**Fig. S1a**).

While a solved co-crystal structure is required to decisively determine the effect of each residue, we hypothesize that since Fc residue 434 is located near FcRn residue L135 in the crystal structure, introduction of W likely increases the overall hydrophobicity of the interaction. Further, Fc residue 311 is located next to H310, in proximity to FcRn residues E115 and E116. Thus, introduction of R311 is likely to engage in and strengthen the hydrogen bond network with FcRn residues E115, E116 and possibly even E133.

In contrast to R311 and W434, Fc residue 428 is located further away from the interaction interface and its substitution to E likely contributes via indirect effects. Introduction of E in position 428 of the Fc may potentially introduce hydrogen bond pairing with H435 which in turn could stabilize Fc loop 433-436 under acidic pH conditions. Such an effect of E428 when combined with R311 and W434 is supported by FcRn binding data showing that back-substitution of this residue to M428 disrupts the pH dependency of the interaction (**Fig. S1b-c**). An indirect contribution of H435 to FcRn binding is supported by previous crystallographic data showing that this residue only engages in a weak π:π interaction with FcRn main chain residue D130, even though its substitution to A abrogates FcRn binding[21].

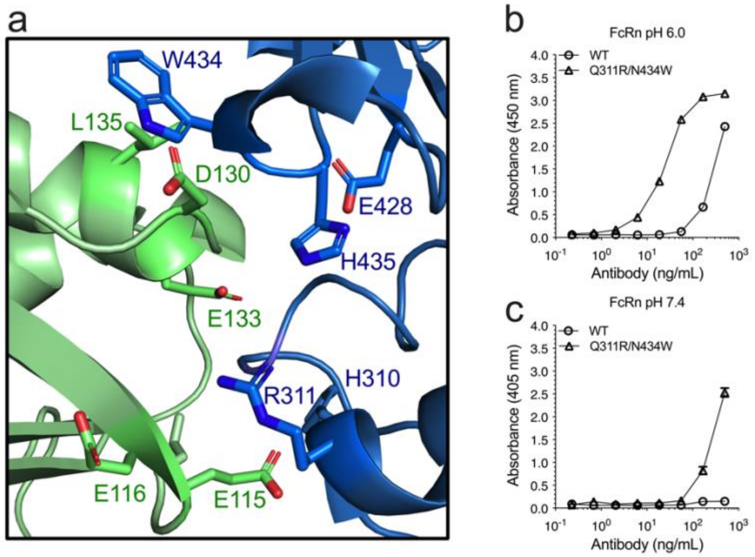

Figure S1. Rationale for enhanced pH dependent FcRn binding. (**a**) Close-up of the core interaction interface in the solved co-crystal structure of truncated recombinant human FcRn (green) in complex with IgG1 Fc (blue) (PDB entry 4NOU[21]. The REW amino acid substitutions (R311/E428/W434) were introduced into the IgG1 Fc using PyMOL. Further, the Fc residues H310 and H435 and FcRn residues E115, E116, E133, D130 and L135 are shown. (**b-c**) ELISA showing binding of anti-NIP IgG1 WT and Q311R/N434W to truncated recombinant FcRn at pH 6.0 and 7.4. Shown as mean±s.d. of duplicates.

SI Text 2:

To evaluate the effect of the REW substitutions on production rate, we produced anti-NIP IgG1 WT and REW in Expi293 cells and the amounts of secreted protein in filtered cell culture supernatant were quantified using an antigen specific ELISA (**Fig. S2a**). The results showed that both antibodies were produced equally well (~100 mg/L) (**Fig. S2b**). Structural integrity was evaluated by non-reducing SDS-PAGE and analytical SEC, which showed that both anti-NIP IgG1 WT and REW migrated to their expected molecular weights (**Fig. S2c**) and eluted as monomeric fractions during analytical SEC (**Fig. S2d-f**). Thermal stability was tested using either dye-based (Sypo Orange) or differential scanning fluorimetry (DSF) using a Lightcycler instrument or by label-free nanoDSF using a Prometheus nanoDSF instrument. Both methods showed that the REW substitutions did not alter the Tm (°C) compared to WT IgG1 (**Fig. S2g-i**). The nanoDSF analysis further showed that the onset of protein aggregation coincided with the Tm (°C) of both antibodies by measuring dynamic light scattering (DLS) (**Fig. S2j**). Next, mapping of the N297-linked *N*-glycans of NIP WT and REW using liquid chromatography tandem mass spectrometry (LC-MS/MS) (**Fig. S2k**) revealed highly similar profiles, except from a minor fucose and galactose increase in REW within the five most prevalent *N*-glycan forms detected (**Fig. S2l, Table S1**). Overall, the main glycoforms of both antibodies were comparable to those found in natural IgG with high fucosylation, low bisection, intermediate galactosylation and low sialyation[80].

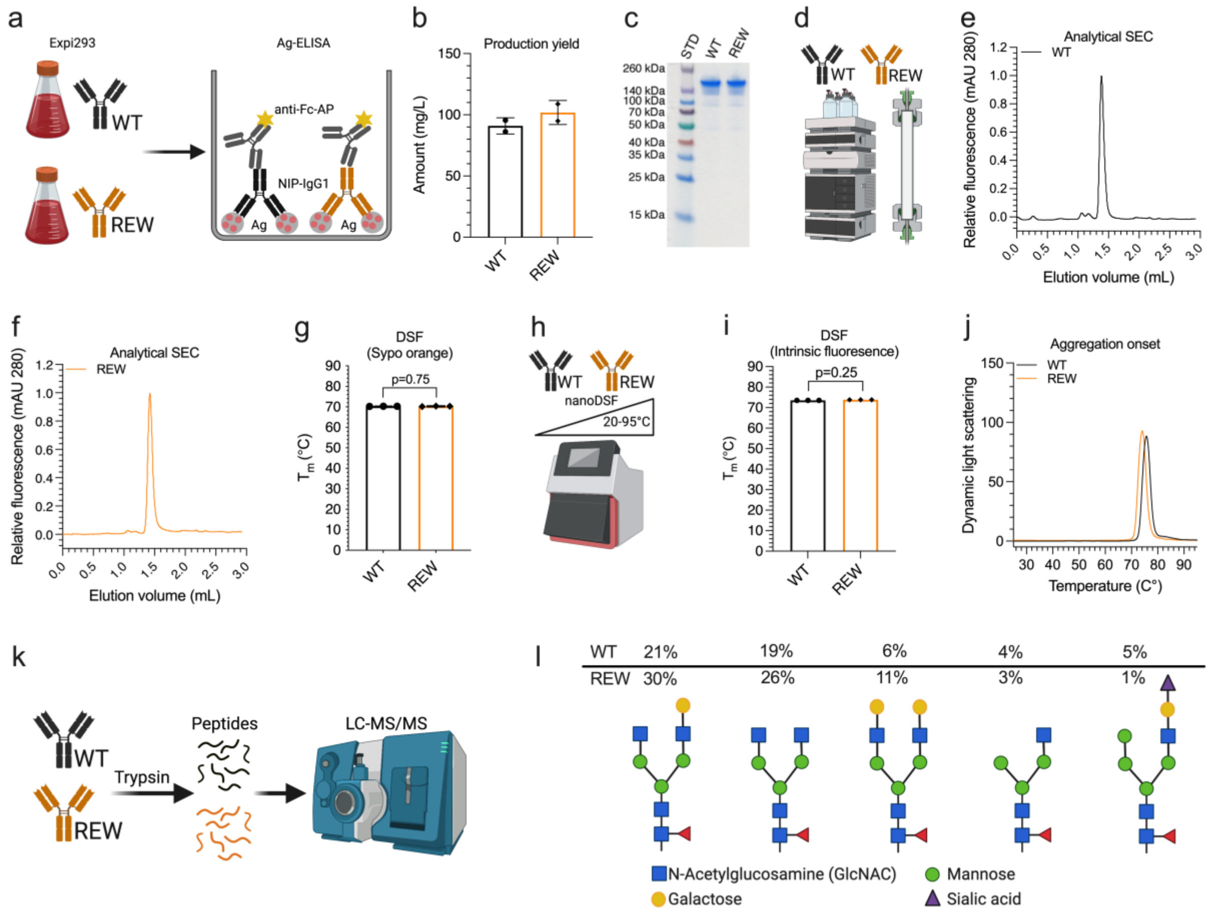

Figure S2. Production, thermal stability, and *N*-glycosylation mapping. (**a**) Illustration showing the Expi293 system, and the antigen specific ELISA used to compare expression levels of anti-NIP IgG1 WT and REW in cell culture supernatant. (**b**) Production yield (mg/mL) of anti-NIP IgG1 WT and REW in Expi293 cells, shown as mean±s.d of supernatant from one cell culture analyzed in duplicates. (**c**) Non-reducing SDS-PAGE showing migration of affinity purified anti-NIP IgG1 WT and REW. (**d-f**) Illustration of and analytical SEC elution profiles of affinity and SEC purified anti-NIP IgG1 WT and REW. (**g**) Tm (°C) of anti-NIP IgG1 WT and REW determined using dye-based DSF (sypo orange) and (**h-i**) illustration and Tm (°C) of anti-NIP IgG1 WT and REW determined using label free nano-DSF, shown as mean±s.d of triplicates. (**j**) Aggregation onset of anti-NIP IgG1 WT and REW as a function of temperature (°C) measured by nanoDSF, shown as mean±s.d of triplicates. (**k-l**) Illustration of N297-glycan mapping procedure by LC-MS/MS and representation showing the percentages of the five most prevalent N297-linked *N*-glycans in anti-NIP IgG1-WT and REW. (g and i) Paired Wilcoxon t-test. Source data are provided as a Source Data file. (a, d, h, k, and l) were created using BioRender.com.

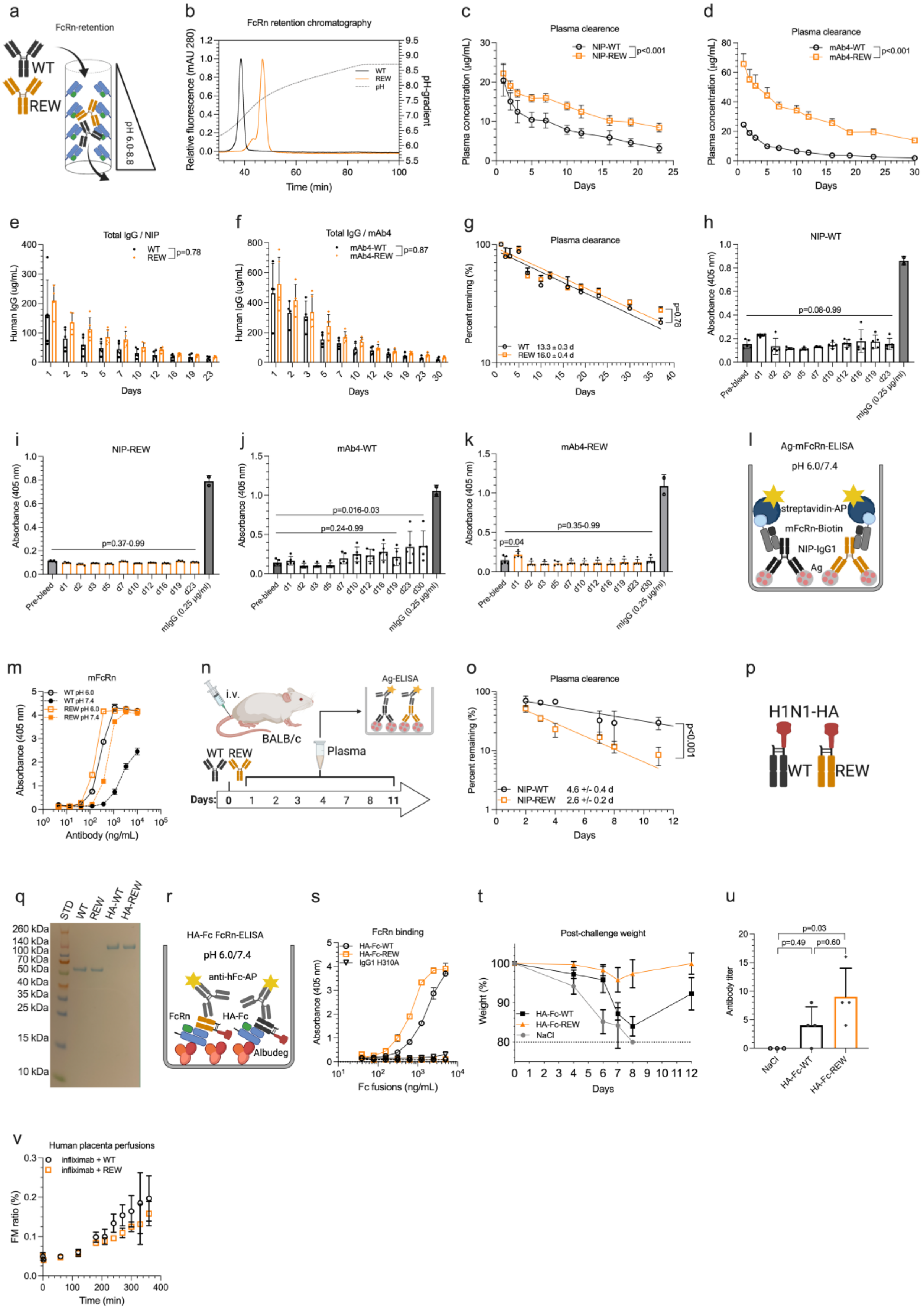
a
FcRn-retention
WT
REW
pH 6.0-8.8
b
FcRn retention chromatography
Relative fluorescence (mAU 280)
pH-gradient
Time (min)
WT
REW
pH
c
Plasma clearence
NIP-WT
NIP-REW
p<0.001
Plasma concentration (µg/mL)
Days
d
Plasma clearence
mAb4-WT
mAb4-REW
p<0.001
Plasma concentration (µg/mL)
Days
e
Total IgG / NIP
WT
REW
p=0.78
Human IgG (ug/mL)
Days
f
Total IgG / mAb4
mAb4-WT
mAb4-REW
p=0.87
Human IgG (ug/mL)
Days
g
Plasma clearance
Percent reminng (%)
WT 13.3 ± 0.3 d
REW 16.0 ± 0.4 d
p=0.78
Days
h
NIP-WT
Absorbance (405 nm)
p=0.08-0.99
Pre-bleed
mIgG (0.25 µg/ml)
i
NIP-REW
Absorbance (405 nm)
p=0.37-0.99
j
mAb4-WT
Absorbance (405 nm)
p=0.016-0.03
p=0.24-0.99
k
mAb4-REW
Absorbance (405 nm)
p=0.35-0.99
p=0.04
l
Ag-mFcRn-ELISA
pH 6.0/7.4
streptavidin-AP
mFcRn-Biotin
NIP-IgG1
Ag
m
mFcRn
WT pH 6.0
WT pH 7.4
REW pH 6.0
REW pH 7.4
Absorbance (405 nm)
Antibody (ng/mL)
n
i.v.
BALB/c
Ag-ELISA
Plasma
WT REW
Days:
o
Plasma clearence
Percent remaining (%)
NIP-WT 4.6 +/- 0.4 d
NIP-REW 2.6 +/- 0.2 d
p<0.001
Days
p
H1N1-HA
WT
REW
q
STD
WT
REW
HA-WT
HA-REW
260 kDa
140 kDa
100 kDa
70 kDa
50 kDa
40 kDa
35 kDa
25 kDa
15 kDa
10 kDa
r
HA-Fc FcRn-ELISA
pH 6.0/7.4
anti-hFc-AP
FcRn
HA-Fc
Albudeg
s
FcRn binding
HA-Fc-WT
HA-Fc-REW
IgG1 H310A
Absorbance (405 nm)
Fc fusions (ng/mL)
t
Post-challenge weight
HA-Fc-WT
HA-Fc-REW
NaCl
Weight (%)
Days
u
p=0.03
p=0.49
p=0.60
Antibody titer
NaCl
HA-Fc-WT
HA-Fc-REW
v
Human placenta perfusions
infliximab + WT
infliximab + REW
FM ratio (%)
Time (min)

Figure S3. FcRn binding, plasma concentration, immunogenicity and biodistribution. (**a-b**) Illustration of human FcRn retention chromatography showing elution of anti-NIP IgG1 WT and REW from the column through a pH gradient (pH 6.0 – 8.8), shown as relative fluorescence units (mAU 280 nm). (**c**) Plasma concentrations of anti-NIP IgG1 WT and REW in IVIg pre-loaded (500 mg/kg) Tg32 mice (n=5 animals per group). (**d**) Plasma concentrations of anti-SARS-CoV-2 (mAb4) IgG1 WT and REW in IVIg pre-loaded (500 mg/kg) Tg32 mice (n=5 animals per group). (**e**) Human IgG concentrations (anti-NIP + IVIg) in plasma samples from Tg32 mice (n=5 animals per group). (**f**) Human IgG plasma concentrations (mAb4 + IVIg) in plasma samples from Tg32 mice (n=5 animals per group). (**g**) Plasma clearance of anti-NIP IgG1 WT and REW (5 mg/kg) in Tg32 mice without IVIg pre-load, shown as mean±s.d of percent antibody remaining in plasma over time (n=5 animals per group). (**h**) Immunogenicity ELISAs showing reactivity of endogenous mouse IgG to anti-NIP IgG1 WT, (**i**) anti-NIP IgG1 REW, (**j**) mAb4 IgG1-WT and (**k**) mAb4 IgG1-REW compared to pre-bleed samples (n=5 animals per group). Mouse IgG was included as a positive control to verify reactivity of the detection antibody. (**l**) Illustration of the ELISA set-up for (**m**) binding of anti-NIP IgG1 WT and REW to mouse FcRn (mFcRn) at pH 6.0 and 7.4, shown as mean±s.d of duplicates. (**n**) Experimental overview and (**o**) plasma clearance of anti-NIP IgG1 WT and REW in WT Balb/c mice, shown as mean±s.d of percent antibody remaining in plasma over time (n=5 animals per group). (**p**) Illustration of H1N1 HA-fused monovalent to either a WT or REW IgG1 Fc fragment and (**q**) non-reducing SDS-PAGE showing migration of HA-WT and HA-REW Fc-fusions compared to unfused WT and REW Fc. (**r**) Illustration of the ELISA set-up for (**s**) binding of HA-WT and HA-REW Fc fusions to human FcRn at pH 6.0 (solid lines) and pH 7.4 (stippled lines), shown as mean±s.d of duplicates. (**t**) Average post-challenge weight of animals in groups of Tg32 mice vaccinated with HA-WT (n=4 animals per group) (black), HA-REW (n=5 animals per group) (orange) Fc fusions or NaCl control (n=4 animals per group) (grey), shown as mean±s.e.m (**u**) HA-specific antibody titers in mice vaccinated with HA-Fc-WT, HA-Fc-REW or NaCl. (**v**) Maternal to fetal transport ratio (F/M ratio) of infliximab (anti-TNFα; IgG1) in presence of either anti-NIP IgG1 WT or REW, shown as mean±s.d (n=4 placentas per group). (c, d, e, f, g, and o) RM Two-way ANOVA with Šídák's multiple comparison test, (h, i, j, and k) one-way ANOVA with Dunnett`s multiple comparison test and (u) Kruskal-Wallis test. Source data are provided as a Source Data file. (a, l, n, p, and r) were created using BioRender.com.

SI Text 3

To provide a rationale for enhanced on-target C1q engagement through more efficient formation of Fc:Fc contacts, we used a model of a hexameric IgG1 assembly from the solved crystal structure of the full length anti-HIV antibody b12 (PDB entry 1HZH)[40, 79] with a close-up of the Fc-Fc interface where the REW substitutions are located (**Fig. S4a**). Regarding R311 in the C_H2 domain, we could not identify any clear potential interaction residues on the adjacent Fc. In contrast, W434 is situated close to Y436 on the adjacent Fc, and the two residues likely engage in an aromatic stacking interaction. Furthermore, the indole moiety of the W434 aromatic ring may form a π-cation interaction with E428. This hypothesis is supported by ELISA C1q binding data showing that back-substitution of R311 to Q311 (EW) had no effect on C1q binding while back-substitution of R311 to Q311 and E428 to M428 (N434W) only had a minor impact on C1q binding (**Fig. S4b**). This identifies W434 as main driver of enhanced on-target C1q binding and complement activation of the REW variant.

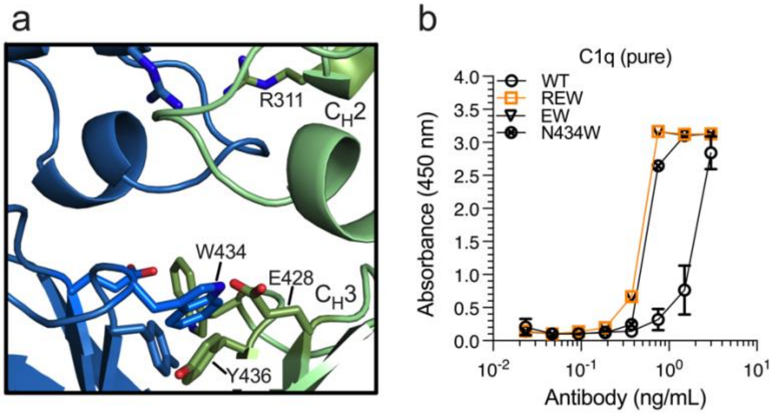

Figure S4. Rationale for enhanced Fc:Fc contact formation of REW. (**a**) Close-up of the Fc:Fc interface of a hexameric assembly of IgG1 molecules focusing on the REW substitutions. One Fc is shown in blue while the adjacent Fc is shown in green. The figure was made in PyMOL. (**b**) ELISA showing binding of antigen captured anti-NIP IgG1 WT, REW, M428E/N434W and N434W to pure human C1q, shown as mean$\pm$s.d of duplicates.

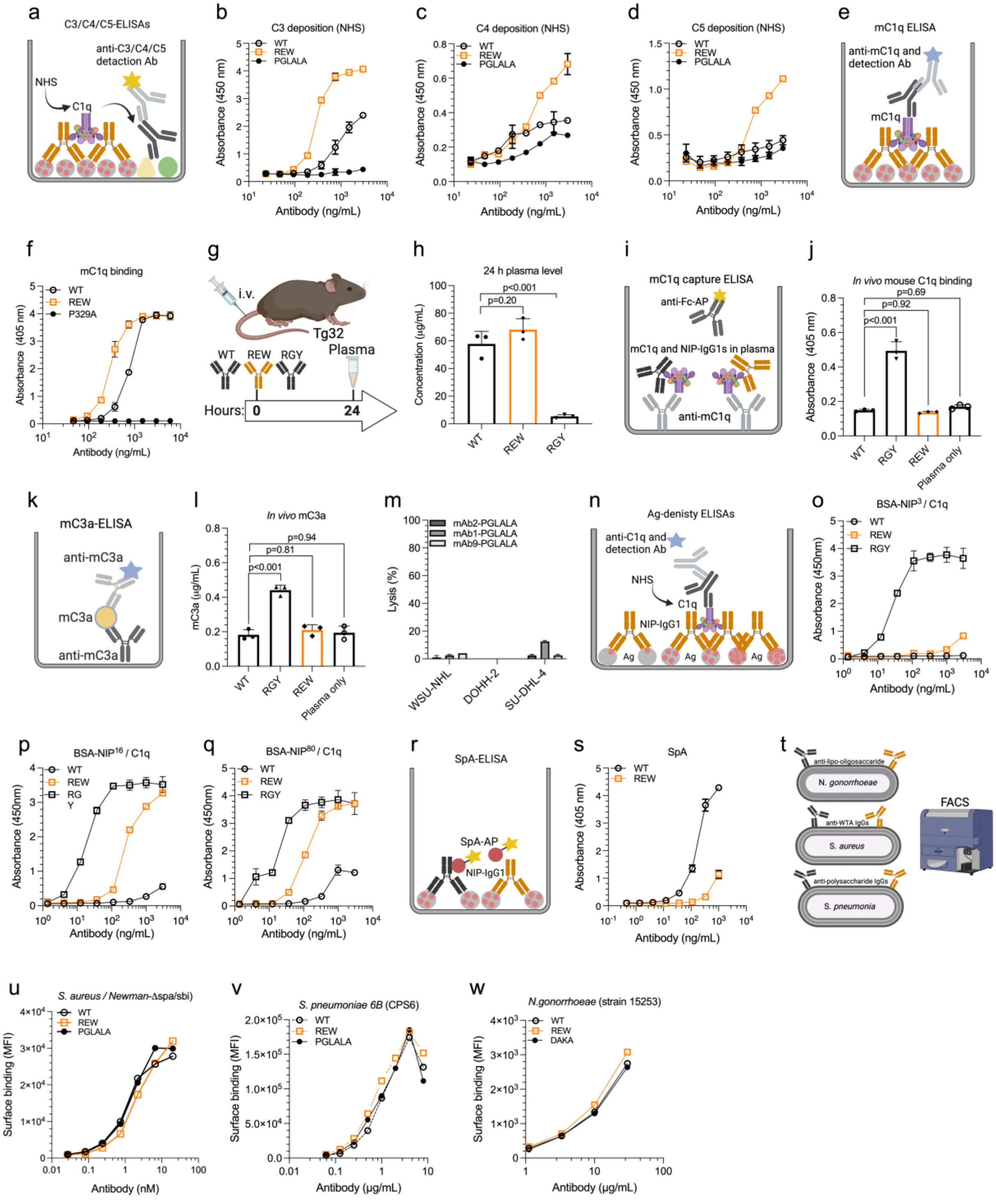

a
C3/C4/C5-ELISAs
anti-C3/C4/C5 detection Ab
NHS
C1q
b
C3 deposition (NHS)
WT
REW
PGLALA
Absorbance (450 nm)
Antibody (ng/mL)
c
C4 deposition (NHS)
WT
REW
PGLALA
Absorbance (450 nm)
Antibody (ng/mL)
d
C5 deposition (NHS)
WT
REW
PGLALA
Absorbance (450 nm)
Antibody (ng/mL)
e
mC1q ELISA
anti-mC1q and detection Ab
mC1q
f
mC1q binding
WT
REW
P329A
Absorbance (405 nm)
Antibody (ng/mL)
g
i.v.
Tg32
WT
REW
RGY
Plasma
Hours:
0
24
h
24 h plasma level
p<0.001
p=0.20
Concentration (µg/mL)
WT
REW
RGY
i
mC1q capture ELISA
anti-Fc-AP
mC1q and NIP-IgG1s in plasma
anti-mC1q
j
In vivo mouse C1q binding
p=0.69
p=0.92
p<0.001
Absorbance (405 nm)
WT
RGY
REW
Plasma only
k
mC3a-ELISA
anti-mC3a
mC3a
anti-mC3a
l
In vivo mC3a
p=0.94
p=0.81
p<0.001
mC3a (µg/mL)
WT
RGY
REW
Plasma only
m
mAb2-PGLALA
mAb1-PGLALA
mAb9-PGLALA
Lysis (%)
WSU-NHL
DOHH-2
SU-DHL-4
n
Ag-denisty ELISAs
anti-C1q and detection Ab
NHS
C1q
NIP-IgG1
Ag
Ag
Ag
o
BSA-NIP3 / C1q
WT
REW
RGY
Absorbance (450nm)
Antibody (ng/mL)
p
BSA-NIP16 / C1q
WT
REW
RGY
Absorbance (450nm)
Antibody (ng/mL)
q
BSA-NIP80 / C1q
WT
REW
RGY
Absorbance (450nm)
Antibody (ng/mL)
r
SpA-ELISA
SpA-AP
NIP-IgG1
s
SpA
WT
REW
Absorbance (405 nm)
Antibody (ng/mL)
t
anti-lipo-oligosaccaride
N. gonorrhoeae
anti-WTA IgGs
S. aureus
anti-polysaccharide IgGs
S. pneumonia
FACS
u
S. aureus / Newman-Δspa/sbi)
WT
REW
PGLALA
Surface binding (MFI)
Antibody (nM)
v
S. pneumoniae 6B (CPS6)
WT
REW
PGLALA
Surface binding (MFI)
Antibody (µg/mL)
w
N.gonorrhoeae (strain 15253)
WT
REW
DAKA
Surface binding (MFI)
Antibody (µg/mL)

Figure S5. Complement activity, antigen density, SpA and bacterial surface binding by antibody variants. (**a**) Illustration of ELISA assays and deposition of (**b**) C3, (**c**) C4 and (**d**) C5 from NHS in presence of titrated amounts of antigen captured anti-NIP IgG1 WT, REW and PGLALA, shown as mean±s.d of duplicates. (**e-f**) Illustration of the ELISA set-up for binding of titrated amounts of antigen captured anti-NIP IgG1 WT, REW and P329A to pure mouse C1q. (**g**) Illustration of the animal experiment to determine *in vivo* complement activation in the presences of anti-NIP IgG1 WT, REW and RGY. (**h**) Plasma levels of anti-NIP IgG1 WT, REW and RGY 24 h post i.v, administration (10 mg/kg) (n=3). (**i-j**) Illustration of the ELISA set-up for binding of anti-NIP IgG1 WT, REW and RGY in concentration normalized plasma to mouse C1q (n=3 animals per group) (**k-l**) Illustration of the anti-mouse C3a ELISA set-up and detection of mouse C3a in concentration normalized plasma samples from mice injected with anti-NIP IgG1 WT, REW and RGY or plasma only (n=3 animals per group). (**m**) Calcein-AM release assay showing CDC activity of anti-CD20 mAb2 (low CDC), mAb1 (intermediate CDC) and mAb9 (high CDC) IgG1 PGLALA variants against lymphoma cell lines WSU-NHL (low CD20), DOHH-2 (intermediate CD20) and SU-DHL-4 (high CD20), shown as mean±s.d of duplicates. (**n**) Illustration of ELISA assay and binding of C1q to anti-NIP IgG1 WT, REW and RGY variants captured on equimolar amounts of BSA conjugated to (**o**) 3, (**p**) 16 or (**q**) 80 NIP-hapten molecules, shown as mean±s.d of duplicates. (**r**) Illustration of ELISA assay and (**s**) binding of S. aureus SpA to antigen captured anti-NIP IgG1 WT and REW, shown as mean±s.d of duplicates. (**t**) Illustration of FACS surface binding assays and binding of (**u**) anti-WTA (clone 4497) IgG1 WT, REW and PGLALA to Newman-ΔspA/sbi *S. aureus*, (**v**) binding of anti-capsule polysaccharide IgG1 WT, REW and PGLALA to *S. pneumoniae* serotype 6B and (**w**) binding of anti-gonococcal lipo-oligosaccharide (clone 2C7) IgG1 variants to *N. gonorrhoeae* (strain 15253), shown as mean fluorescent intensity (MFI). (h, j, and l) One-way ANOVA with Dunnetts multiple comparisons test. Source data are provided as a Source Data file. (a, e, g, i, k, n, r, and t) were created using BioRender.com.

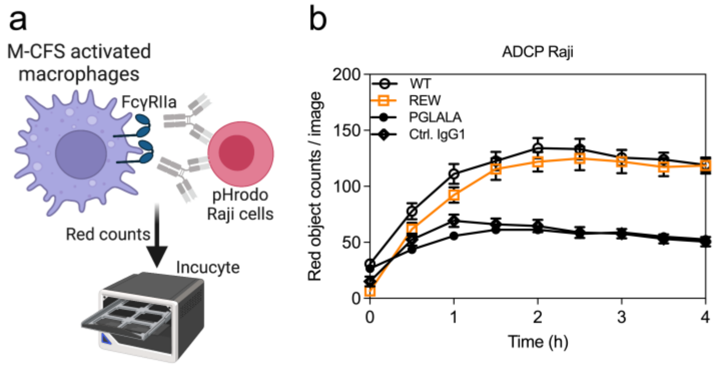

Figure S6. ADCP. (**a**) Illustration of the ADCP assay and (**b**) macrophage mediated ADCP of Raji target cells by mAb2 anti-CD20 IgG1 WT, REW, PGLALA and Ctrl IgG1, shown as mean±s.e.m from 5 replicates performed in parallel. Source data are provided as a Source Data file. (a) were created using BioRender.com.

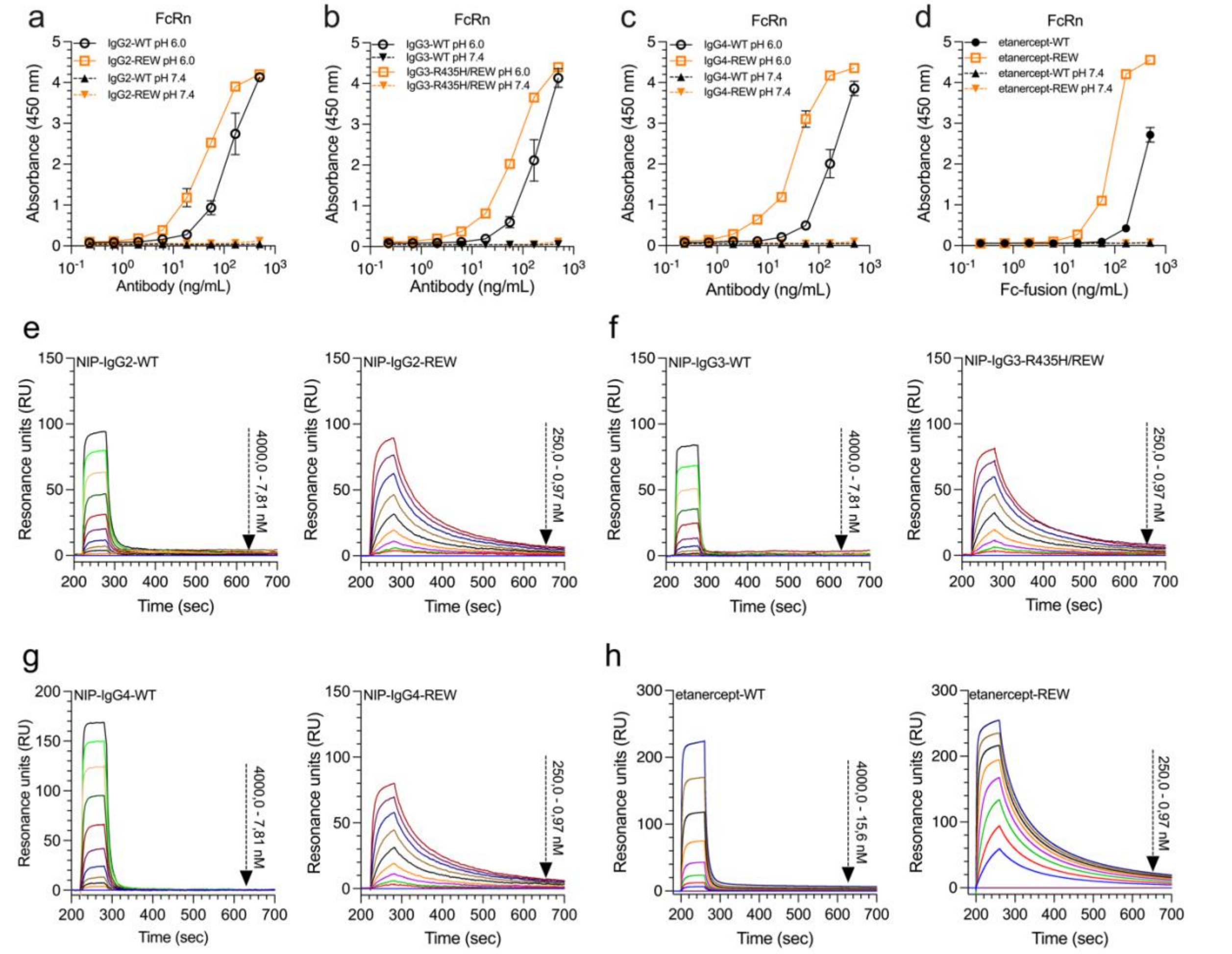

Figure S7. Binding of REW-engineered IgG subclasses and etanercept to human FcRn. (**a-d**) ELISAs showing binding of titrated amounts of antigen captured anti-NIP IgG2 WT and REW, IgG3 WT and R435H/REW (RH/REW), (**c**) IgG4 WT and REW and (**d**) recombinant etanercept WT and REW to human FcRn at pH 6.0 and 7.4, shown as mean±s.d of duplicates. SPR sensorgrams showing reversible binding of anti-NIP (**e**) IgG2-WT and REW, (**f**) IgG3 WT and IgG3-R435H/REW, (**g**) IgG4 WT and REW and (**h**) recombinant etanercept WT and REW human FcRn at pH 6.0. The experiments were performed at 25°C at a flow rate of 50 μl/min. Source data are provided as a Source Data file.

SI Text 4

To assess how the REW substitutions affects Rf+ binding, we made recombinant WT and REW IgG1 Fc fragments and coated equal amounts in ELISA, which was controlled by measuring binding to a polyclonal anti-human IgG1 Fc specific detection antibody (**Fig. S8a-b**). To measure binding of Rf antibodies, Rf+ human serum was added to ELISA plates containing the immobilized Fc fragments followed by detection using a pan-IgG light chain specific antibody (**Fig. S8c**). The results showed reduced reactivity of Rf+ serum towards REW than WT, while antibodies in NHS did not bind (**Fig. S8d**).

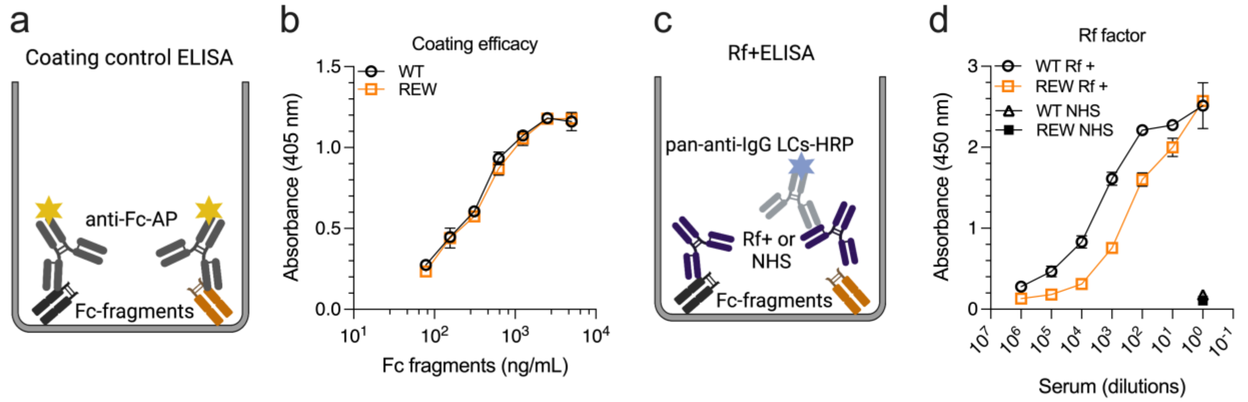

Figure S8. REW reduces binding to Rf+ factor. (**a**) Illustration showing ELISA assay and (**b**) coating levels of recombinant WT and REW IgG1 Fc fragments using an AP-conjugated anti-human IgG Fc antibody, shown as mean±s.d of duplicates. (**c**) Illustration of ELISA assay and (**d**) binding of antibodies from Rf+ human serum or NHS to WT and REW IgG1 Fc fragments, shown as mean±s.d of duplicates. Source data are provided as a Source Data file. (a and c) were created using BioRender.com.

SI Text 5:
To delineate the FcRn binding properties of REW we performed a side-by-side in-vitro interaction analysis with established IgG1 variants engineered for altered FcRn binding and plasma half-life. To do so, we first produced anti-NIP IgG1 YTE[24], DHS[25], LS[23], KF[36] and YTE/KF[81] and compared binding of biotinylated human FcRn to anti-NIP IgG1 WT and REW in ELISA (**Fig. S9a**). The results revealed a receptor binding hierarchy at pH 6.0 of YTE/KF>REW>KF>LS>YTE>DHS>WT and a hierarchy at pH 7.4 of YTE/KF>KF>LS>YTE>REW>DHS>WT (**Fig. S9b-e**). The observed binding hierarchy at pH 6.0 was confirmed by SPR binding kinetics in experiments run in parallel with anti-NIP IgG1 WT and REW in Fig. 1f-g, Table S2 (**Fig. S9f-j, Table S8**). Likewise, the binding hierarchy at pH 7.4 observed in ELISA was confirmed in SPR by injecting 4000 nM FcRn over high levels of immobilized antibody variants (2000 RU) (**Fig. S9k-l**). FcRn retention chromatography experiments run in parallel with the data presented in Fig. S3b, Table S3 (anti-NIP IgG1 WT and REW) showed the same elution pH hierarchy as in ELISA and SPR (**Fig. S9m, Table S9**). Taken together these data places REW in the high binding range at acidic pH (6.0) and in the low binding range at neutral pH (7.4). We then performed a side-by-side comparison of the plasma half-life of anti-NIP IgG1 WT, REW (Fig. 1n) and the DHS variant, the latter recently shown to have considerably extended half-life in human FcRn / human IgG / human FcγR expressing mice[25], using the IVIg pre-loaded Tg32 mouse model. The results revealed similar plasma clearance profiles for REW (14.2 ± 2.3 days) and DHS (15.4 ± 2.7 days) (**Fig. S9n**).

Further, we compared the complement activity of REW to that of the IgG1 variant E430G[43] using recombinant ofatumumab anti-CD20 IgG1 variants to measure CDC against “hard-to-kill” Raji lymphoma cells. Using a ^{51}Cr release assay (**Fig. S9o**), we observed equal enhanced target cell killing of REW and E430G over WT (**Fig S9p, Table S10**). When binding of C1q to antigen captured anti-NIP IgG1 REW was compared to that of other established Fc-engineering strategies for altered FcRn binding and half-life, REW bound the strongest (**Fig. S9q**).

Next, no major differences in binding to high affinity FcγRI were observed between WT and REW or the half-life extended IgG1 variants LS, YTE and DHS when captured on their cognate antigen in ELISA (**Fig S10a-b**). In contrast, more efficient binding of REW was observed to all human low affinity FcγRs compared to WT, LS, YTE and DHS variants in the same ELISA set-up (**Fig. S10c-h**). Notably, strongly reduced binding was observed for YTE against all receptors, in line with published data[9, 27].

To determine whether the increased binding of REW to the low affinity FcγRs was a result of altered affinity or an effect of target binding and potentially formation of Fc:Fc contacts, we compared the binding profiles of anti-NIP IgG1 WT and REW to the human FcγRs in the absence of antigen using SPR (**Fig. S10i**). This showed highly overlapping binding profiles to all receptors (**Fig. S10j-p**). For REW, a slightly slower on-rate and off-rate were observed for FcγRIIa-H131 and FcγRIIa-R131 as well as a slightly slower on-rate and faster off-rate for FcγRI.

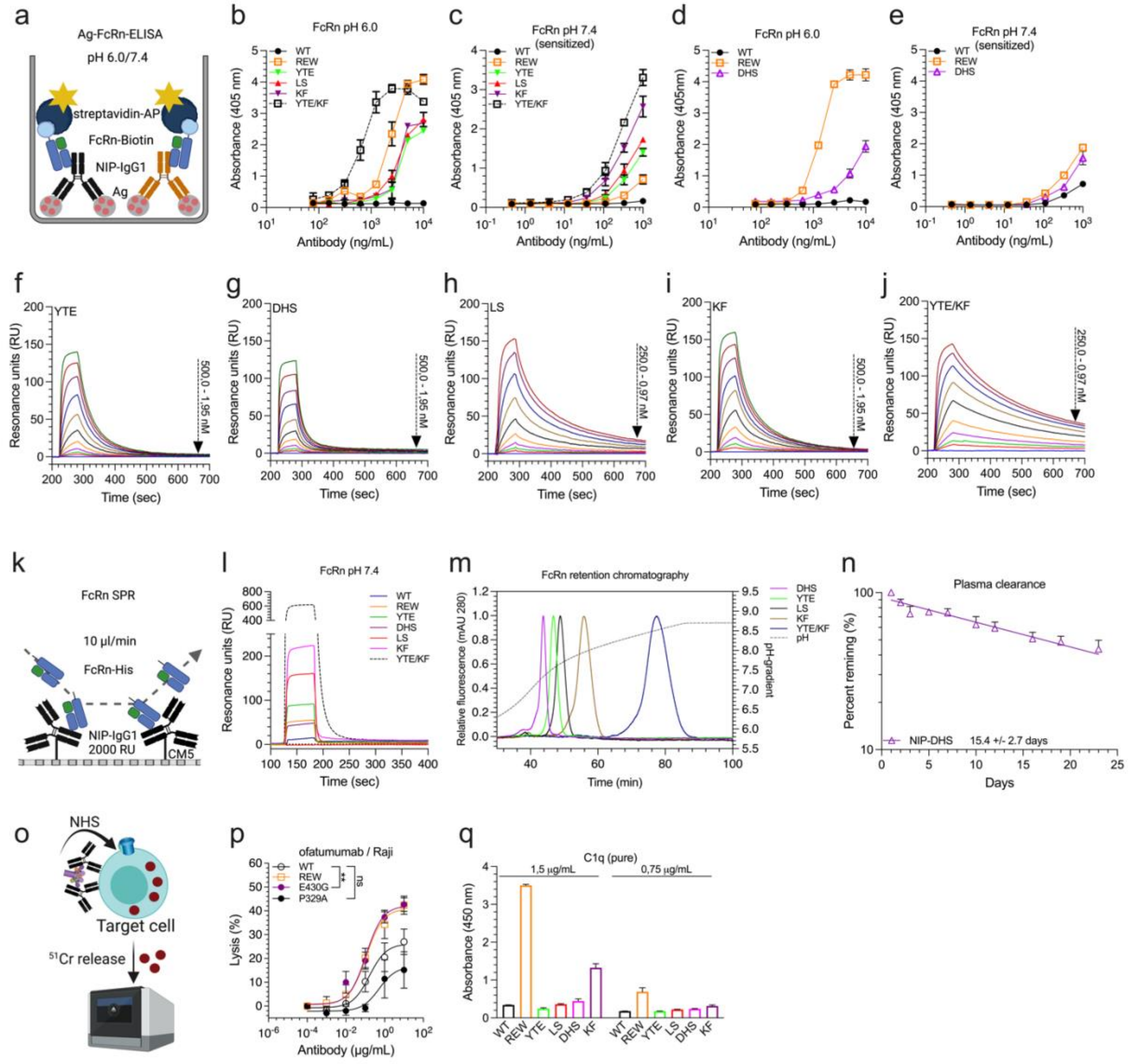

Figure S9. pH dependent FcRn binding, plasma clearance and complement activity of engineered IgG1 variants. (**a**) Illustration of ELISA assay and (**b-c**) binding of titrated amounts of anti-NIP IgG1 WT, REW, YTE, LS, KF and YTE/KF or (**d-e**) anti-NIP IgG1 WT, REW and DHS to biotinylated FcRn at pH 6.0 or pH 7.4. Biotinylated FcRn were pre-incubated with streptavidin-AP at pH 7.4 to sensitize the assay, shown as mean$\pm$s.d of duplicates. (**f-j**) SPR sensorgrams showing binding (RU) of anti-NIP YTE, DHS, LS, KF and YTE/KF at pH 6.0. The SPR experiments were performed in parallel with the experiments in Fig. 1f-g and the data is directly comparable. (**k**) Illustration of SPR assay at pH 7.4 and (**l**) SPR sensorgram showing binding (RU) of FcRn (4000 nM) to high levels of immobilized anti-NIP IgG1 variants (2000 RU) at pH 7.4. The experiments were performed at 25°C with a flow rate of 50 μl/min at pH 6.0 and 10 μl/min at pH 7.4. (**m**) FcRn retention chromatography showing elution of anti-NIP IgG1 YTE, DHS, LS, KF and YTE/KF through a pH gradient (pH 6.0 – 8.8). Elution peaks have been normalized to relative fluorescence units (mAU 280 nm), the experiment was performed in parallel with that in Fig. S3b and the data is directly comparable. (**n**) Plasma clearance of NIP IgG1 DHS following i.v. administration (5 mg/kg) in IVIg pre-loaded (500 mg/kg) Tg32 mice, shown as mean$\pm$s.d of percent antibody remaining in plasma over time (n=5 animals per group), the experiment was performed in parallel with that in Fig. 1n and the data is directly comparable. (**o**) Illustration of ^{51}Cr release assay and (**p**) CDC activity of ofatumumab (anti-CD20) WT, REW, E430G and P329A against Raji lymphoma cell lines. Shown as

mean±s.d of four replicates performed in parallel. (**q**) ELISA showing binding of pure human C1q to antigen captured anti-NIP IgG1 WT, REW, YTE, LS, DHS and KF, shown as mean±s.d of duplicates. (p) Two-way ANOVA with Dunnett`s multiple comparison test. Source data are provided as a Source Data file. (a, k, and o) were created using BioRender.com.

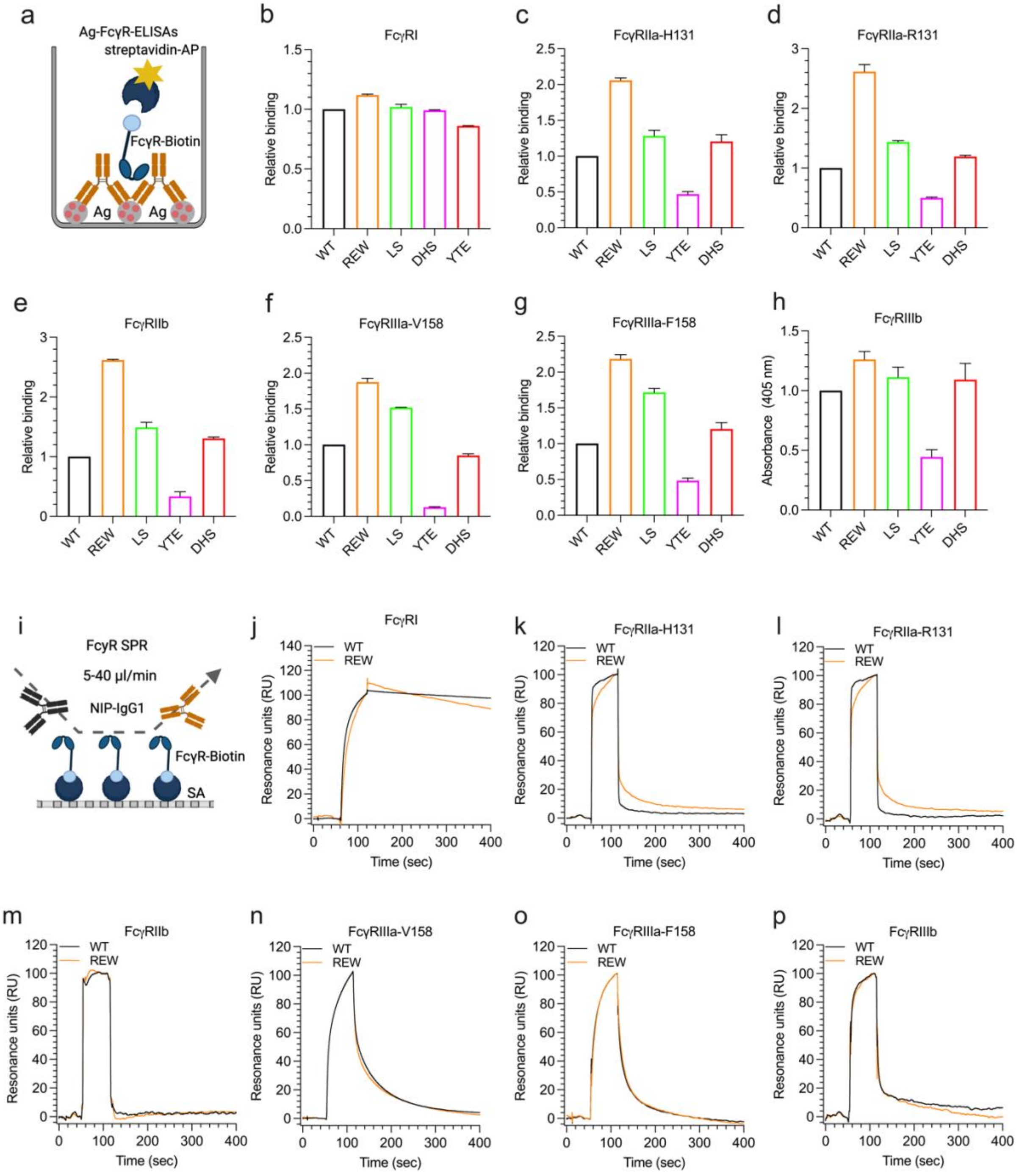

Figure S10. Human FcγR binding properties of Fc-engineered IgG1 variants. (**a**) Illustration of the ELISA set-up used to compare binding of WT anti-NIP IgG1 and the Fc-engineered variants REW, LS, YTE and DHS to human FcγRs. (**b-h**) ELISA binding of antigen captured anti-NIP IgG1 WT, REW, LS, YTE and DHS to FcγRI, FcγRIIa-H131, FcγRIIa-R131, FcγRIIb, FcγRIIIa-V158, FcγRIIIa-F158 and FcγRIIIb. Data shown as binding relative to anti-NIP IgG1 WT, mean±s.d of duplicates. Biotinylated FcγRIIIb was pre-incubated with streptavidin-AP to sensitize detection of binding. (**i**) Illustration of the SPR set-up for (**j-p**) binding of single concentrations of anti-NIP IgG1 WT and REW to FcγRI, FcγRIIa-H131, FcγRIIa-R131, FcγRIIb, FcγRIIIa-V158, FcγRIIIa-F158 and FcγRIIIb. The maximum binding response was

normalized to 100 RU for clarity. Source data are provided as a Source Data file. (a and i) were created using BioRender.com.

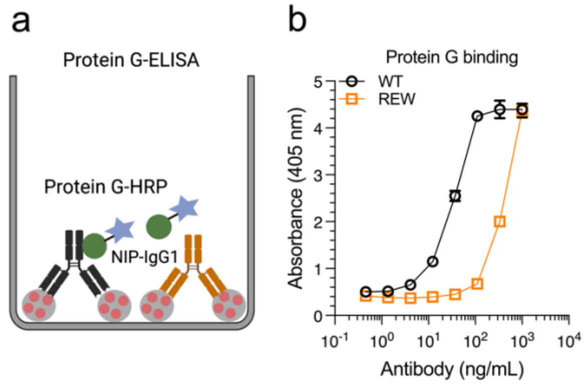

Figure S11. REW reduces protein G binding. (**a**) Illustration of ELISA assay and (**b**) binding of antigen captured anti-NIP IgG1 WT and REW to *Streptococcus* Protein G, shown as mean±s.d of duplicates. Source data are provided as a Source Data file. (a) were created using BioRender.com.

Table S1. Mapping of N297-linked *N*-glycans in WT and REW NIP specific IgG1 variants. Table showing the relative amounts of N297-linked *N*-glycans in IgG1 WT and REW. GlcNac = Acetylglucosamine, Hex = Mannose/Galactose, Fuc = Fucose and SiA = Sialic Acid.

N297-linked glycan form	Abundance (%) WT	Abundance (%) REW
4GlcNac-4Hex-1Fuc	20.6	30.5
4GlcNac-3Hex-Fuc	18.9	26.2
4GlcNac-5Hex-1Fuc	6.1	11.4
2GlcNac-5Hex	5.7	2.4
3GlcNac-3Hex-1Fuc	5.6	6.2
3GlcNac-5Hex-1Fuc-1SiA	5.5	0.9
3GlcNac-5Hex-1Fuc	5.1	1.4
3GlcNac-4Hex-1Fuc	3.7	2.7
4GlcNac-5Hex-1Fuc-1SiA	1.7	2.0
3GlcNac-6Hex-1Fuc-1SiA	2.2	0.5
4GlcNac-5Hex-1Fuc	1.2	0.1
4GlcNac-4Hex-1Fuc-1SiA	0.5	0.4
3GlcNac-6Hex-1SiA	0.8	1.8
3GlcNac-5Hex-1SiA	0.4	0.1
3GlcNac-4Hex-1Fuc-1SiA	0.9	0.2
5GlcNac-5Hex-1Fuc	0.3	0.5
5GlcNac-4Hex-1Fuc	1.9	3.1
3GlcNac-6Hex-1Fuc	2.3	0.7
5GlcNac-3Hex-1Fuc	0.7	0.9
3GlcNac-6Hex	0.9	0.4
3GlcNac-5Hex	1.2	0.4
2GlcNac-5Hex-1Fuc	0.3	0.0
4GlcNac-3Hex	0.3	0.6
3GlcNac-4Hex	0.3	0.2
2GlcNac-4Hex-1Fuc	1.0	0.2
2GlcNac-4Hex	1.5	0.6
2GlcNac-3Hex-1Fuc	1.0	0.7
2GlcNac-3Hex	1.0	0.5
2GlcNAc-2Hex-fuc	0.7	0.7
2GlcNAc-2Hex	0.9	0.3
2GlcNAc-Hex-fuc	0.3	0.3
2GlcNAc-Hex	0.8	0.3
2GlcNAc-fuc	0.0	0.1
2GlcNAc	0.5	0.2
GlcNAc-Fuc	0.1	0.2
GlcNAc	1.6	0.4
peptide	2.6	1.3

Table S2. Human FcRn binding kinetics of IgG Fc engineered variants at pH 6.0.					
IgG1 variant	Ka (10^4/Ms)	Kd (10^{-2}/s)	K_D (nM)	Range	Fold change from WT (K_D)
NIP-WT	48.3	8.25	171.0	4000-15.6	-
NIP-REW	65.3	0.55	8.39	250-0.9	20.4
Kinetic rate constants were determined using a first order (1:1) Langmuir biomolecular interaction model.					

Table S3. Elution pH of NIP IgG1 WT and REW from human FcRn.		
IgG1 variant	Elution range (pH)	Elution peak (pH)
NIP-WT	6.73-7.02	6.95
NIP-REW	7.43-7.67	7.58

Table S4. Non-compartmental PK analysis (NCA)								
IgG1 variant	Dose	Route	AUC (µg*d/mL)	Cmax (µg/mL)	CL (mL/d/kg)	MRT (d)	Vss (mL/kg)	$T_{1/2}$ (days)
NIP-WT	5 mg/kg	IV Bolus	197.6	20.4	21.4	12.2	260.9	7.8
NIP-REW	5 mg/kg	IV Bolus	310.4	22.2	9.4	26.2	245.6	18.2
mAb4-WT	5 mg/kg	IV Bolus	207.9	24.5	22.0	12.9	278.9	10.7
mAb4-REW	5 mg/kg	IV Bolus	904.9	65.6	4.3	21.3	92.9	15.0
AUC = Area under the curve from time 0 to the last measured time point.<br>C_{max} = Maximum concentration following administration.<br>CL = Clearance.<br>MRT = Mean residency time.<br>Vss = Volume of distribution at steady state.<br>$T_{1/2}$ = Terminal plasma half-life.								

Table S5. EC50 values for CDC killing of Newman-ΔspA/sbi s.aureus opsonized with anti-WTA (clone 4497) IgG1 variants.	
IgG1 variant	EC50 (µg/mL)
WT	0.067
REW	0.0063

Table S6. EC50 values for CDC killing of Newman-WT s. aureus opsonized with anti-WTA (clone 4497) IgG1 variants.	
IgG1 variant	EC50 (µg/mL)
WT	0.88
REW	0.19

**Table S7. FcRn binding kinetics of REW IgG subclasses and Fc fusion etanercept.**					
IgG variant	Ka (10^4/Ms)	Kd (10^{-2}/s)	K_D (nM)	Range	Fold change from WT
NIP-IgG2-WT	13.8	5.36	388.0	4000-7.81	
NIP-IgG2-REW	57.0	0.75	13.3	250-0.9	29.2
NIP-IgG4-WT	9.58	12.3	1280.0	4000-7.81	
NIP-IgG4-REW	60.6	0.80	13.1	250-0.9	97.7
NIP-IgG3-WT	33.7	17.7	525.0	4000-7.81	
NIP-IgG3-RH/REW	67.4	0.70	10.3	250-0.9	50.9
etanercept-WT	34.8	12.0	345.6	4000-15.6	
etanercept-REW	130.5	0.14	10.9	250-1.95	31.7
Kinetic rate constants were determined using a first order (1:1) Langmuir biomolecular interaction model.					

**Table S8. Human FcRn binding kinetics of IgG Fc engineered variants at pH 6.0.**					
IgG1 variant	Ka (10^4/Ms)	Kd (10^{-2}/s)	K_D (nM)	Range	Fold change from WT
NIP-DHS	75.6	3.04	40.3	500-1.95	4.2
NIP-YTE	59.4	1.68	28.2	500-1.95	6.0
NIP-LS	42.2	0.58	13.7	250-0.9	12.4
NIP-KF	77.0	1.22	15.9	500-1.95	10.7
NIP-YTE/KF	87.1	0.30	3.44	250-0.9	49.7
Kinetic rate constants were determined using a first order (1:1) Langmuir biomolecular interaction model.					

**Table S9. Elution pH of NIP IgG1 variants from human FcRn.**		
IgG1 variant	Elution range (pH)	Elution peak (pH)
NIP-DHS	7.19-7.43	7.37
NIP-YTE	7.43-7.67	7.57
NIP-LS	7.54-7.78	7.69
NIP-KF	7.83-8.07	8.00
NIP-YTE/KF	8.39-8.65	8.53

**Table S10. EC50 values for CDC activity against Raji cell opsonized by ofatumumab anti-CD20 IgG1 variants using a ^{51}Cr release assay.**	
IgG1 variant	EC50 (µg/mL)
WT	0.16
REW	0.11
E430G	0.10

Table S11. T cell (MHC) epitope prediction of Fc-engineered IgG1 variants using NetMHC4.1.			
IgG1 variants	Number of strong binders	Number of weak binders	Number of peptides
WT	4	5	322
REW	4	5	322
LS	4	5	322
DHS	4	4	322
YTE	3	5	322
KF	4	5	322
YTE/KF	3	5	322
Binding of 9-mer peptides against representative HLA supertypes.<br>Rank threshold for strong binding peptides: 0.5<br>Rank threshold for weak binding peptides: 2.0			

Table S12. Antibody epitope prediction of Fc-engineered IgG1 variants by IEDB analysis.		
IgG1 constant region variants	Number of peptides	Peptide sequences
WT	12	VFPLAPSSKSTSG<br>GALTSGVHTFPAVLQSS<br>PSSSLGTQ<br>KPSNTKVDKKVEPKSCDKTHTCPPCPAPELLGGPSVFLFPPKPKD<br>VSHED<br>HNAKTKPREEQYNS<br>WLN<br>ALPAPIEKTISKAKGQPREP<br>LPPSREEMTKN<br>PENNY<br>SRWQQGNVFSC<br>HNHYTQKSLSLS
REW	13	VFPLAPSSKSTSGG<br>ALTSGVH<br>FPAVLQSS<br>VPSSSLGT<br>KPSNTKVDKKVEPKSCDKTHTCPPCPAPELLGGPSVFLFPPKPKD<br>VSHED<br>VHNAKTKPREEQYNS<br>RDWLNG<br>KALPAPIEKTISKAKGQPREP<br>TLPPSREEMTKN<br>PENNY<br>SRWQQGNVFSC<br>LHWHYTQKSLSLSP
LS	14	VFPLAPSSKSTSG<br>YFP<br>GALTSGVH<br>FPAVLQSS<br>SSSLGTQ<br>KPSNTKVDKKVEPKSCDKTHTCPPCPAPELLGGPSVFLFPPKPK<br>VSHED<br>HNAKTKPREEQYNS<br>WLN<br>KALPAPIEKTISKAKGQPREP<br>LPPSREEMTKN<br>PENNY<br>KSRWQQGNVFS<br>LHSHYTQKSLSLSP

DHS	14	PLAPSSKSTSGG<br>YFP<br>ALTSGVH<br>PAVLQSSG<br>SSSLGTQ<br>KPSNTKVDKKVEPKSCDKTHTCPPCPAPELLGGPSVFLFPPKPK<br>VSH<br>HNAKTKPREEQYNST<br>WLNG<br>ALPAPIEKTISKAKGQPREPQV<br>TLPPSREEMTKN<br>PENNY<br>SRWQQGNVFS<br>LHSHYTQKSLSLSP
YTE	14	VFPLAPSSKSTSGG<br>YFP<br>LTSGV<br>PAVLQSS<br>PSSSLGTQ<br>KPSNTKVDKKVEPKSCDKTHTCPPCPAPELLGGPSVFLFPPKPKDT<br>HNA<br>TKPREEQYNS<br>WLN<br>ALPAPIEKTISKAKGQPREPQV<br>TLPPSREEMTKN<br>PENNY<br>SRWQQGNVFS<br>HNHYTQKSLSLS
KF	13	PLAPSSKSTSG<br>YFP<br>ALTSGVH<br>VLQSS<br>SSSLGTQ<br>KPSNTKVDKKVEPKSCDKTHTCPPCPAPELLGGPSVFLFPPKPKD<br>HNAKTKPREEQYNST<br>WLNG<br>KALPAPIEKTISKAKGQPREPQV<br>TLPPSREEMTKN<br>PENNY<br>SRWQQGNVFS<br>LKFHYTQKSLSLSP
YTE/KF	11	VFPLAPSSKSTSG<br>YFP<br>ALTSGV<br>QSS<br>SSSLGTQ<br>KPSNTKVDKKVEPKSCDKTHTCPPCPAPELLGGPSVFLFPPKPKD<br>VSH<br>ALPAPIEKTISKAKGQPREPQVYTLPPSREEMTKNQ<br>PENNY<br>SRWQQGNVFSC<br>LKFHYTQKSLSLSP
Threshold: 0.5		
