# Supplementary material for: Human IgG Fc-engineering for enhanced plasma half-life, mucosal distribution and killing of cancer cells and bacteria
Source: Nat Commun. 2024 Mar 7;15:2007. doi: 10.1038/s41467-024-46321-9 (PMC10920689; doi:10.1038/s41467-024-46321-9)
Supplement: Supplementary file 3 — Reporting Summary [file 41467_2024_46321_MOESM3_ESM.pdf]

nature portfolio

Corresponding author(s): Jan Terje Andersen

Last updated by author(s): Jan 20, 2024

Reporting Summary

Nature Portfolio wishes to improve the reproducibility of the work that we publish. This form provides structure for consistency and transparency in reporting. For further information on Nature Portfolio policies, see our Editorial Policies and the Editorial Policy Checklist.

Statistics

For all statistical analyses, confirm that the following items are present in the figure legend, table legend, main text, or Methods section.

n/a	Confirmed	
[ ]	[x]	The exact sample size (*n*) for each experimental group/condition, given as a discrete number and unit of measurement
[ ]	[x]	A statement on whether measurements were taken from distinct samples or whether the same sample was measured repeatedly
[ ]	[x]	The statistical test(s) used AND whether they are one- or two-sided<br>*Only common tests should be described solely by name; describe more complex techniques in the Methods section.*
[x]	[ ]	A description of all covariates tested
[x]	[ ]	A description of any assumptions or corrections, such as tests of normality and adjustment for multiple comparisons
[ ]	[x]	A full description of the statistical parameters including central tendency (e.g. means) or other basic estimates (e.g. regression coefficient) AND variation (e.g. standard deviation) or associated estimates of uncertainty (e.g. confidence intervals)
[ ]	[x]	For null hypothesis testing, the test statistic (e.g. *F*, *t*, *r*) with confidence intervals, effect sizes, degrees of freedom and *P* value noted<br>*Give P values as exact values whenever suitable.*
[x]	[ ]	For Bayesian analysis, information on the choice of priors and Markov chain Monte Carlo settings
[x]	[ ]	For hierarchical and complex designs, identification of the appropriate level for tests and full reporting of outcomes
[x]	[ ]	Estimates of effect sizes (e.g. Cohen's *d*, Pearson's *r*), indicating how they were calculated

Our web collection on statistics for biologists contains articles on many of the points above.

Software and code

Policy information about availability of computer code

Data collection	Open source software:<br>gPKPDsim (doi: 10.1007/s10928-017-9562-9)<br>-Microsoft Excel version 16.79.1
Data analysis	-Graphpad Prism version 10.1.0<br>-BIAevaluation software version 4.1<br>-MatLab R2021a<br>-gPKPDsim (doi: 10.1007/s10928-017-9562-9)<br>-PyMOL version 2.5.3

For manuscripts utilizing custom algorithms or software that are central to the research but not yet described in published literature, software must be made available to editors and reviewers. We strongly encourage code deposition in a community repository (e.g. GitHub). See the Nature Portfolio guidelines for submitting code & software for further information.

Data

Policy information about availability of data

All manuscripts must include a data availability statement. This statement should provide the following information, where applicable:

- Accession codes, unique identifiers, or web links for publicly available datasets
- A description of any restrictions on data availability
- For clinical datasets or third party data, please ensure that the statement adheres to our policy

Publicly available data sets used:
Protein database accession number 4NOU.
Protein database accession number 1HZH.

Research involving human participants, their data, or biological material

Policy information about studies with human participants or human data. See also policy information about sex, gender (identity/presentation), and sexual orientation and race, ethnicity and racism.

Reporting on sex and gender	The ex vivo human placental perfusion study involves the use of human biological material. Sex was considered during study design and only pregnant female participants were enrolled since they are the only sex that have placentas. Sex was determined based on assignment. Gender was not considered as part of the study design.<br><br>As only females participated in the study disaggregated data for sex and gender was not collected.<br><br>Sex or gender was not considered during collection of human blood samples prior to isolation of human immune cells used for cellular assays.
Reporting on race, ethnicity, or other socially relevant groupings	Race, ethnicity or other social groupings were not considered as part of the study design.
Population characteristics	To minimize variation in the ex vivo placenta perfusion model, mothers who smoked, had diabetes or other pregnancy complications were excluded from the study.
Recruitment	Placentas from uncomplicated pregnancies resulting in vaginal or caesarean section were donated by women givin birth at Copenhagen University Hospital. Informed consent was obtained in accordance with the Declaration of Helsinki.<br><br>Human blood samples were collected following informed consent at Utrecht Medical Center or University Medical Center Schleswig-Holstein.<br><br>No self-selection bias or other biases have been recorded.
Ethics oversight	- Ethical commitee of the Communities of Copenhagen and Fredriksberg and the Danish Data Protection Agency.<br>- Medical Research Ethics Committee NedMec.<br>- Ethics Committee of Kiel University.

Note that full information on the approval of the study protocol must also be provided in the manuscript.

Field-specific reporting

Please select the one below that is the best fit for your research. If you are not sure, read the appropriate sections before making your selection.

☒ Life sciences ☐ Behavioural & social sciences ☐ Ecological, evolutionary & environmental sciences

For a reference copy of the document with all sections, see nature.com/documents/nr-reporting-summary-flat.pdf

Life sciences study design

All studies must disclose on these points even when the disclosure is negative.

Sample size	Sample is mainly relevant to 1) animal experiments, 2) Human placenta perfusion experiments and 3) cellular experiments.<br>Sample size in animal experiments were determined based on ethical perspectives (the 3 Rs) and previously published and collected data confirming the sufficiency of the used sample size for robust analysis and statistical analysis.<br>Animal experiments: PMID: 35982144, PMDI: 35118359, PMID: 29391560.<br>Human placenta perfusion experiment: PMID: 35981406, PMID: 23843496.<br>Cellular experiments: PMID29391560, PMID: 26718855.
Data exclusions	Data was excluded from further interpretation if found to be below the sensitivity threshold of the analytical method or identified as outlayers

	using Grubb`s test. This occurred only in a limited subset of data points and had no impact on the conclusions.
Replication	Analysis of samples from in vivo studies were performed twice using established methodology and reviewed by internal and external experts. Cellular experiments were performed 2 or 3 times as independent experiments using 2-4 technical replicates. Biochemical experiments were repeated at least once and analyzed using appropriate evaluation software. All stated instrumentation underwent routinely maintenance and quality assessment both prior to and during the data collection period. All attempts at replication were successful.
Randomization	Not relevant to the study, as it would compromise ability to interpret data.
Blinding	Irrelevant to our study, as it would compromise the ability to interpret data.

Reporting for specific materials, systems and methods

We require information from authors about some types of materials, experimental systems and methods used in many studies. Here, indicate whether each material, system or method listed is relevant to your study. If you are not sure if a list item applies to your research, read the appropriate section before selecting a response.

Materials & experimental systems

n/a	Involved in the study	
☐	☒	Antibodies
☐	☒	Eukaryotic cell lines
☒	☐	Palaeontology and archaeology
☐	☒	Animals and other organisms
☒	☐	Clinical data
☒	☐	Dual use research of concern
☒	☐	Plants

Methods

n/a	Involved in the study	
☒	☐	ChIP-seq
☒	☐	Flow cytometry
☒	☐	MRI-based neuroimaging

Antibodies

Antibodies used

Antibodies approved for clinical use (Tradename; Common name; Manufacturer;:
-privigen, IVIg, CSL Behring.
-Remicade, infliximab, Janssen Biologics.
-Enbrel, etanercept, Pfizer.

Details for commercial antibodies used in experimental analyses (Tradename; Supplier, Product number and Lot number):
-Goat anti-GST antibody, Rockland Immunochemicals, 200-301-200, 24891.
-Mouse IgG from serum, Sigma, I5381,1003399528.
-Anti-Mouse IgG (Fc-specific)-Alkaline Phosphatase antibody produced in goat, Sigma, A9316, 058M4837V.
-Anti-Human IgG (Fc-specific)-Alkaline Phosphatase antibody produced in goat , Sigma, A9544, 088M4799V.
-Anti-Human IgG (Fc-specific) produced in goat, Sigma, I2136, 0000154675.
-Rabbit anti-Human C1q, Dako/Agilent, A0136 ,20019896.
-Rabbit anti-Human complement component C3, Dako/Agilent, A0062, 056.
-Rabbit anti-Human complement component C4, Dako/Agilent, F0169, 058.
-Rabbit anti-Human complement component C5, Dako/Agilent, A0056, 0000609E.
-Donkey anti-Rabbit IgG HRP-linked, Cytiva, NA934, 17212129.
-Goat anti-Mouse C1q Polyclonal Antiserum, Creative Biolabs, CTA-P-023, C8L073023MM02.
-Rabbit anti-Goat IgG AP-conjugate, Merck, 12-448, 000014321.
-Mouse anti-Human C9C5b, Diatech Monoclonals, DIA 011-01, Q0919-01.
-Rabbit Pan anti-Human IgG light chains, RevMab Biosciences, 32-1031-00, S-03-02117.

Validation

Goat anti-GST antibody, Rockland Immunochemicals, 200-301-200:
Citations: PMID: 29507658, PMID: 26867008, PMID: 23817741.
CiteAb reference: https://www.citeab.com/antibodies/1907826-200-301-200-anti-gst-mouse-monoclonal-antibody-2?des=9c0341a21e004f13

Mouse IgG from serum, Sigma, I5381:
Citations: PMID: 17869502, PMID: 17586580, PMID: 27514757, PMID: 9616171.
CiteAb reference: https://www.citeab.com/antibodies/10354000-i5381-igg-from-mouse-serum?des=9856ef707b992568

Anti-Mouse IgG (Fc-specific)-Alkaline Phosphatase antibody produced in goat, Sigma, A9316:
Quality level: MQ200 (https://www.sigmaaldrich.com/NO/en/life-science/quality-and-regulatory-management/m-clarity-program)
Citations: PMID: 15162468, PMID: 21471663.
CiteAb reference: https://www.citeab.com/antibodies/1514945-a9316-anti-mouse-igg-whole-molecule-alkaline-phosp?

des=4c84723d443a8c36

Anti-Human IgG (Fc-specific)-Alkaline Phosphatase antibody produced in goat , Sigma, A9544:
Quality level: MQ300 (https://www.sigmaaldrich.com/NO/en/life-science/quality-and-regulatory-management/m-clarity-program)
Citations: PMID: 11468739, PMID: 27339099, PMID: 35982144, PMID: 35118359, PMID: 29434196, PMID: 26962230.
CiteAb reference: https://www.citeab.com/antibodies/1507096-a9544-anti-human-igg-fc-specific-alkaline-phosphat?des=c31427613b756250

Anti-Human IgG (Fc-specific) produced in goat, Sigma, I2136:
Quality level: MQ300 (https://www.sigmaaldrich.com/NO/en/life-science/quality-and-regulatory-management/m-clarity-program)
Citations: PMID: 10573085, PMID: 28620050, PMID: 28421663.
CiteAb reference: https://www.citeab.com/antibodies/1507041-i2136-anti-human-igg-fc-specific-antibody-produced?des=5094f919fd3afbe7

Rabbit anti-Human C1q, Dako/Agilent, A0136:
Manufacturer website: Traces of contaminating antibodies have been removed by solid-phase absorption.
Citations: PMID: 25904551.

Rabbit anti-Human complement component C3, Dako/Agilent, A0062:
Citations: PMID: 25904551, PMID: 7024314.

Rabbit anti-Human complement component C4, Dako/Agilent, F0169:
Manufacturer website: Traces of contaminating antibodies have been removed by solid-phase absorption with human plasma proteins. Reacts with C4, C4b and C4c but not with C4d. Only reactivity with C4 in crossed immunoelectrophoresis in human plasma.
Citations: PMID: 25904551.
CiteAb reference: https://www.citeab.com/antibodies/3382886-f0169-c4c-complement?des=7befedb1c695abb8

Rabbit anti-Human complement component C5, Dako/Agilent, A0056:
Citations: PMID: 25904551.

Donkey anti-Rabbit IgG HRP-linked, Cytiva, NA934:
Citations: PMID: 25904551.
CyteAb reference: https://www.citeab.com/antibodies/3288289-na934-1ml-amersham-ecl-rabbit-igg-hrp-linked-whole.

Goat anti-Mouse C1q Polyclonal Antiserum, Creative Biolabs, CTA-P-023:
CiteAb reference: https://www.citeab.com/antibodies/15179312-cta-p-023-goat-anti-mouse-c1q-polyclonal-antiserum?des=768aea0a7d9baec1

Rabbit anti-Goat IgG AP-conjugate, Merck, 12-448:
Manufacturer website: This Goat anti-rabbit IgG antibody, Alkaline Phosphatase conjugate is validated for use in ELISA, WB, IH for the detection of Rabbit IgG.
Quality level: MQ100 (https://www.sigmaaldrich.com/NO/en/life-science/quality-and-regulatory-management/m-clarity-program)
Citations: PMID 25904551.
CiteAb reference: https://www.citeab.com/antibodies/1474085-12-448-goat-anti-rabbit-igg-antibody-alkaline-phosp?des=34f44ea72f708cd9

Mouse anti-Human C9C5b, Diatech Monoclonals, DIA 011-01:
Citations: PMID: 3685888, PMID: 4035298, PMID: 21401574, PMID: 8238252.
https://www.citeab.com/antibodies/308436-bpd-dia-011-01-complement-component-c5b-9-ae11?des=da680dc2c92b113c

Rabbit Pan anti-Human IgG light chains, RevMab Biosciences, 32-1031-00:
Manufacturer website: The antibody reacts to both kappa and lambda light chains of human immunoglobulins. It does not react to monkey (Cyno or Rhesus) IgG, mouse IgG, rat IgG, or goat IgG. Validated for ELISA, IH and FC.
CiteAb reference: https://www.citeab.com/antibodies/12722494-32-1031-00-anti-human-ig-light-chains-rabbit-monocl?des=8c322be64863449a

Eukaryotic cell lines

Policy information about cell lines and Sex and Gender in Research

Cell line source(s)	Human embryonic kidney 293E (HEK293E), ATCC, CRL-1573.<br>Carnaval, DSMZ GmbH, ACC 724.<br>Raji, ATCC, CCL-86.<br>WSU-NHL, DSMZ GmbH, ACC 58.<br>DOHH-2, DSMZ GmbH, ACC 47.<br>SU-DHL-4, DSMZ GmbH, ACC 495.<br>T84, ATCC, CCL-248.<br>- HMEC-1-HA-FcRn-EGFP; Boston Children's Hospital, Harvard Medical School and Harvard Digestive Diseases Center, USA; generation of described in doi: 10.1091/mbc.E13-04-0174.<br>- MDCK-hFcRn; Roche Pharma Research and Early Development.<br>-Expi293, ThermoFisher, A14527.
Authentication	Authentication of cell lines:<br>- HEK293E; visual inspection of morphology, proliferation rate monitored, protein production rates of in-house standardized antibody variants monitored.<br>-Expi293; visual inspection of morphology, proliferation rate monitored, protein production rates of in-house standardized antibody variants monitored.<br>-Carnaval, Raji, WSU-NHL, DOHH2, SU-DHL-4; visual inspection of morphology, proliferation rate monitored.<br>T84; visual inspection of morphology, proliferation rate monitored, FcRn expression validated by RT-PCR.<br>- HMEC1-HA-FcRn-EGFP; visual inspection of morphology, proliferation rate monitored, FcRn expression validated by EGFP expression using FACS, cellular recycling of FcRn-negative antibody monitored.<br>- MDCK-hFcRn; visual inspection of morphology, proliferation rate monitored, polarization rate and level monitored; transcellular transport of FcRn-negative antibody monitored.
Mycoplasma contamination	All cell lines tested negative for mycoplasma contamination.
Commonly misidentified lines<br>(See ICLAC register)	No commonly misidentified cell lines were used in this study.

Animals and other research organisms

Policy information about studies involving animals; ARRIVE guidelines recommended for reporting animal research, and Sex and Gender in Research

Laboratory animals	-Hemizygous B6.Cg-Fcgrttm1Dcr Tg(FCGRT)32Dcr/DcrJ mice, The Jackson Laboratory, male and female, age 7-9 weeks.<br>-Homozygous B6.Cg-Fcgrttm1Dcr Tg(FCGRT)32Dcr/DcrJ mice, The Jackson Laboratory, male and female, age 8-10 weeks.<br>-Balb/c wild-type mice, Taconic Farms, male and female, 8 weeks old.
Wild animals	The study did not involve wild animals.
Reporting on sex	All animal experiments were performed using a mix of male and female aged 7-10 weeks, with a weight of 20-30 g/mouse and 3, 5 or 6 mice per group.<br><br>Disaggregated data for sex was not collected.
Field-collected samples	No field collected samples were used in the study.
Ethics oversight	-Animal Care and Use Committee at The Jackson Laboratory<br>-Norwegian Food Safety Authority.

Note that full information on the approval of the study protocol must also be provided in the manuscript.

Plants

Seed stocks	Not relevant to the study.
Novel plant genotypes	Not relevant to the study.
Authentication	Not relevant to the study.
